# Mantle-flow diversion beneath the Iranian plateau induced by Zagros’ lithospheric keel

**DOI:** 10.1038/s41598-021-81541-9

**Published:** 2021-02-02

**Authors:** Ayoub Kaviani, Meysam Mahmoodabadi, Georg Rümpker, Simone Pilia, Mohammad Tatar, Faramarz Nilfouroushan, Farzam Yamini-Fard, Ali Moradi, Mohammed Y. Ali

**Affiliations:** 1 Institute of Geosciences, Goethe University, Frankfurt, Germany; 2 International Institute of Earthquake Engineering and Seismology, Tehran, Iran; 3 Department of Earth Sciences-Bullard Labs, University of Cambridge, Cambridge, UK; 4 Faculty of Engineering and Sustainable Development (ATM), University of Gävle, Gävle, Sweden; 5 Department of Geodetic Infrastructure, Lantmäteriet, Gävle, Sweden; 6 Institute of Geophysics, University of Tehran, Tehran, Iran; 7 Department of Earth Sciences, Khalifa University of Science and Technology, Abu Dhabi, UAE

**Keywords:** Solid Earth sciences, Physics

## Abstract

Previous investigation of seismic anisotropy indicates the presence of a simple mantle flow regime beneath the Turkish-Anatolian Plateau and Arabian Plate. Numerical modeling suggests that this simple flow is a component of a large-scale global mantle flow associated with the African superplume, which plays a key role in the geodynamic framework of the Arabia-Eurasia continental collision zone. However, the extent and impact of the flow pattern farther east beneath the Iranian Plateau and Zagros remains unclear. While the relatively smoothly varying lithospheric thickness beneath the Anatolian Plateau and Arabian Plate allows progress of the simple mantle flow, the variable lithospheric thickness across the Iranian Plateau is expected to impose additional boundary conditions on the mantle flow field. In this study, for the first time, we use an unprecedented data set of seismic waveforms from a network of 245 seismic stations to examine the mantle flow pattern and lithospheric deformation over the entire region of the Iranian Plateau and Zagros by investigation of seismic anisotropy. We also examine the correlation between the pattern of seismic anisotropy, plate motion using GPS velocities and surface strain fields. Our study reveals a complex pattern of seismic anisotropy that implies a similarly complex mantle flow field. The pattern of seismic anisotropy suggests that the regional simple mantle flow beneath the Arabian Platform and eastern Turkey deflects as a circular flow around the thick Zagros lithosphere. This circular flow merges into a toroidal component beneath the NW Zagros that is likely an indicator of a lateral discontinuity in the lithosphere. Our examination also suggests that the main lithospheric deformation in the Zagros occurs as an axial shortening across the belt, whereas in the eastern Alborz and Kopeh-Dagh a belt-parallel horizontal lithospheric deformation plays a major role.

## Introduction

**Figure 1:**
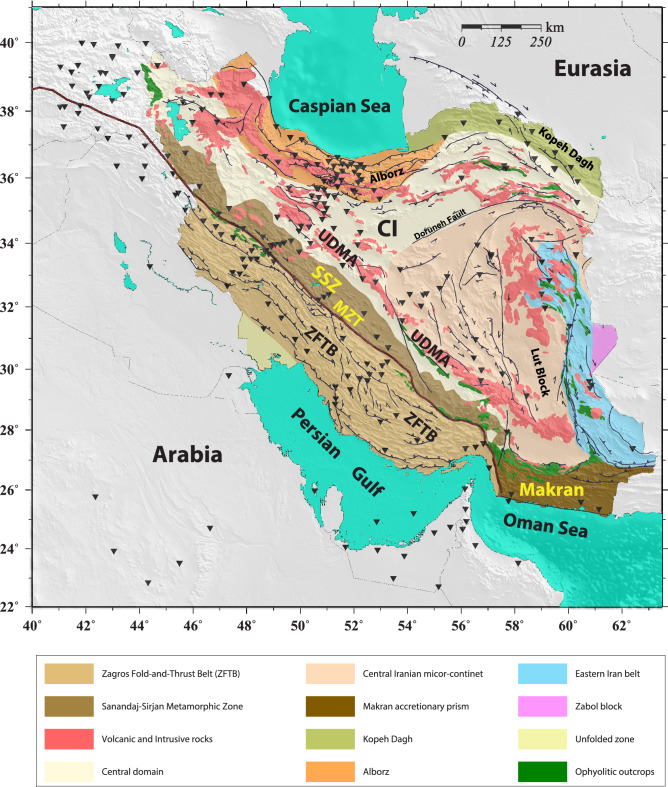
Geological map of Iran and locations of the seismic stations. *ZFTB* Zagros folded-and-thrust belt, *SSZ* Sanandaj-Sirjan metamorphic zone, *UDMA* Urumieh-Dokhtar magmatic assemblage, *MZT* Main Zagros Thrust, *CI* Central Iran. Black inverted triangles indicate the location of seismic stations used in this study. Map is generated using the Generic Mapping Tools (GMT)^60^.

The knowledge about the relationship and causal link between deep mantle processes and surface tectonic features such as mountain building is essential to our understanding of dynamic evolution of continental lithospheres. The Iranian Plateau and Zagros, as a young continental collision zone along the Alp-Himalayan orogenic belt, offer an excellent location to examine such a causal link. Composed of several accreted Gondwanan terranes, the Iranian Plateau was formed as a result of the continental collision between the Arabian and Eurasian plates (Fig. 1). The Zagros collision zone comprises three main provinces: (1) the Zagros Fold-and-Thrust Belt (ZFTB), (2) the Sanandaj-Sirjan metamorphic zone (SSZ), and (3) the Urumieh-Dokhtar Magmatic Assemblage (UDMA). The Arabia-Eurasia oblique convergence, at a rate of ~ 22 mmyr^−1^^1^, has been accommodated by crustal shortening in different orogenic belts (Zagros, Alborz and Kopeh-Dagh) and several strike-slip fault systems across the Iranian Plateau^2,3^.

Previous studies^4,5^ suggest that the lithospheric thickness is less than 120 km beneath the Iranian microplate and Arabia, while it increases up to 250 km beneath central and southern Zagros. It is assumed that the thickening of the Arabian lithosphere beneath the Zagros partially accommodates the Arabia-Eurasia convergence^4^. Furthermore, tomographic images^4,6–10^ show high velocities beneath the Zagros and Kopeh-Dagh areas and low velocities beneath central Iran and the Alborz mountains, suggesting a warmer (weaker) lithospheric mantle beneath the inner part of the plateau, which is trapped and squeezed between colder (stronger) lithospheres.

In a previous study, Kaviani et al.^11^ investigated azimuthal anisotropy beneath the Zagros and Iranian Plateau; however, the limited station coverage and short observational time frame did not allow for a detailed investigation of the entire region. Sadeghi-Bagherabadi et al.^12,13^ presented the results of shear-wave splitting (SWS) analysis from a temporary profile of stations across the NW Zagros, NW central Iran and Alborz. These studies show small-scale variations in anisotropic structure along the profile; however, it is difficult to generalize the conclusions deduced from these limited observations to the entire Zagros and the Iranian Plateau. More recently, Rahimzadeh et al.^14^ report shear-wave splitting observations from a limited number of seismic stations in the Makran region in SE Iran, which suggest that seismic anisotropy in this region is mainly affected by simple shear related to the flat subduction of the Arabian slab beneath the Eurasian plate.

These piecewise earlier studies provided evidence for a complex pattern of anisotropy across the vast region of the Iranian Plateau. On the other hand, the high-resolution seismic anisotropy study across the neighboring Turkish-Anatolian Plateau^15^ suggests a relatively simple pattern of seismic anisotropy governed by large-scale mantle flow. The current station coverage across the Iranian Plateau and surrounding regions motivated us to comprehensively examine the causes of the complex pattern of anisotropy in the region. For this purpose, we use an unprecedented data set of waveforms from a network of 245 stations (Fig. 1) with longer than one decade of observations at a large number of stations. We analyzed > 7600 core-refracted seismic shear phases (SKS, SKKS, and PKS, hereafter called XKS) from the network to investigate azimuthal seismic anisotropy across the study area.

## Results

**Figure 2:**
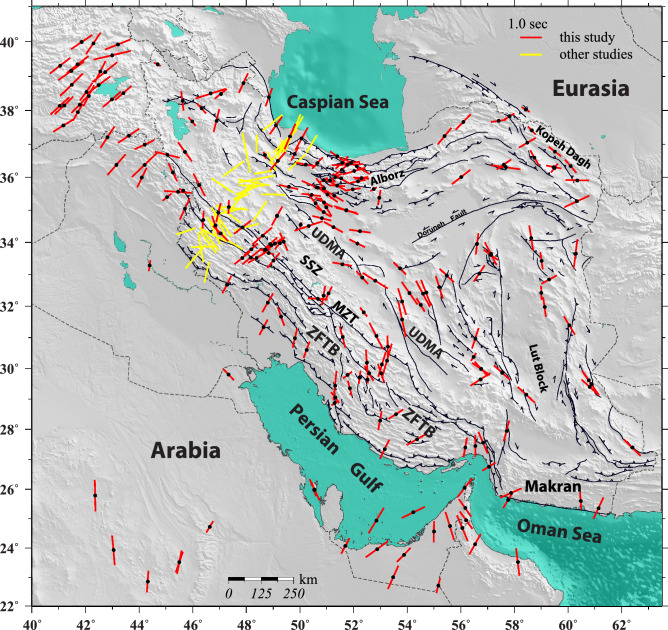
The results of one-layer anisotropic inversion for each station (red bars). Yellow bars illustrate results from previous studies. Each bar is oriented along the fast direction of the one-layer model and its length is proportional to the amount of split time. Map is generated using the GMT^60^.

We jointly analyze the XKS waveforms recorded at each station^16,17^ to calculate the SWS parameters (fast axis and split time) corresponding to a one-layer model of anisotropy beneath the station. In this approach, the splitting parameters of the model is obtained such that they minimize the total energy of the transverse-component (T) of all XKS waveforms recorded at the station. A more detailed description of the approach is given below in the “method” section (refer to Reiss and Rümpker^17^ for further details). In Fig. 2 we show the results of the one-layer inversion at stations where the T-component energy is reduced by more than 30%. The results shown in Fig. 2 are also provided in Table 1 as the supplementary information. In Fig. 2, we also present the results of previous studies (yellow bars)^12,13^. We do not explicitly show earlier results of Kaviani et al.^11^ and Rahimzadeh et al.^14^, since we have re-processed and updated the data at the corresponding stations.

**Figure 3:**
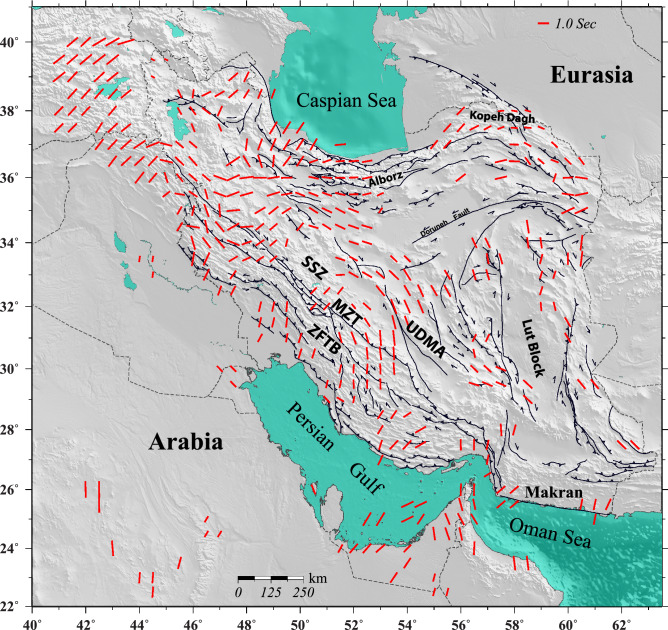
Interpolated anisotropy field at a depth of 150 km as calculated by averaging the directions at individual stations (see text for more explanation). Map is generated using the GMT^60^.

To better identify the characteristic pattern of seismic anisotropy, we resample our observations at grid points separated by 1° in both longitude and latitude. The interpolated anisotropy pattern is shown in Fig. 3. We observe a relatively uniform NE-SW oriented azimuthal anisotropy in the Arabian Plate, eastern Turkey and part of the Zagros, whereas the anisotropy exhibits a more complex pattern across the Iranian Plateau. A striking feature is the gradual change in the direction of anisotropy from a dominantly NNE-SSW trend in the southern Zagros to a dominantly NW–SE trend along a narrow band extending from NW to SE Iran along the Sanandaj-Sirjan metamorphic zone and the Urumieh-Dokhtar magmatic assemblage (Fig. 1). The split time also gradually increases from the Zagros to this narrow band of NW–SE oriented azimuthal anisotropy. The pattern of azimuthal anisotropy turns back to a dominantly NE–SW trend further north and northeast Iran, while a dominantly N–S trend of anisotropy is observed in eastern Iran.

Although the parameters of the one-layer model for each station are obtained by the joint inversion of SKS waveforms, the distribution of individual splitting parameters projected to the piercing points at representative depths of 100 and 200 km (supplementary Figure S1) also highlights the circular pattern of azimuthal anisotropy beneath the Central Zagros (mainly at 200 km depth, Figure S1b). The list of single measurements is provided in the supplementary Table 2.

## Discussion

**Figure 4:**
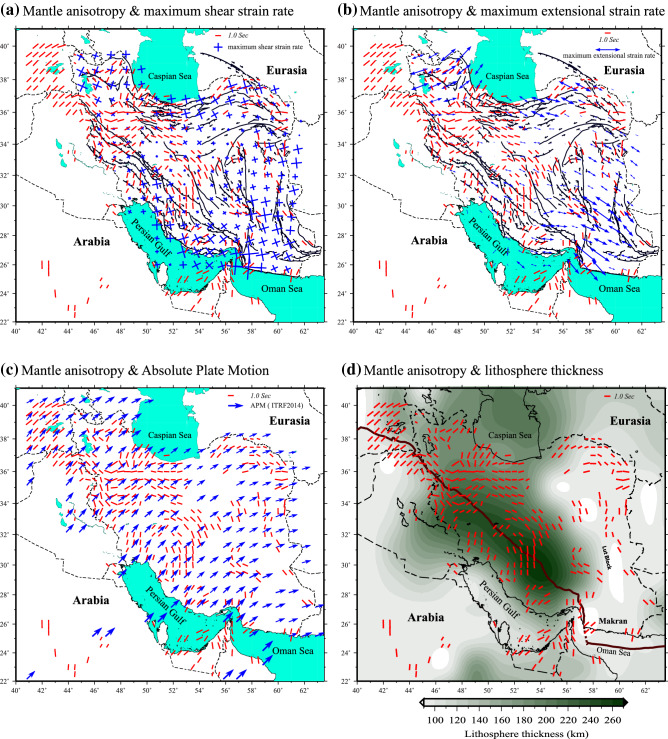
The resampled anisotropy field superimposed on: a) maximum shear strain rate directions, b) maximum extensional strain rate directions, c) the absolute plate motion in the ITRF2014 reference frame^20^ (blue arrows), and d) the lithospheric thickness contour map (from Priestley and McKenzie^5^). GPS velocities and geodetic strain rates are from Khorrami et al^19^. Maps are generated using the GMT^60^.

The main challenge in the interpretation of any SKS splitting observation is the relatively poor depth constraint on the source of the undergoing anisotropy. It is generally assumed that the main source resides in the upper mantle, where the LPO of anisotropic minerals is developed by dislocation creep in crystal lattice^18^. Yet, the discrimination between a lithospheric and/or asthenospheric source of anisotropy is both challenging and crucial when making inferences about the dynamic processes in the upper mantle. Comparison of seismic anisotropy with other surface observations including geological structures, crustal deformation and large-scale plate motion field is a helpful approach to address this ambiguity and make the SWS observation more meaningful. While GPS-derived surface velocity field can provide clues on the plate motion directions and the pattern of the associated large-scale asthenospheric flow (by assuming a coupling of the lithosphere motion to the asthenospheric flow), the lateral gradient of the velocity field, as a measure of surface strain field, can offer insights into the lithospheric deformation (by assuming a vertically coherent deformation). The availability of a relatively uniform and extensive archive of geodetic data across the Iranian Plateau^19^ provides the opportunity to examine the correlation between the pattern of seismic anisotropy, plate motion velocities and surface strain fields. For this purpose, we compare the interpolated anisotropy pattern (red bars) with GPS velocities and horizontal strain rates from Khorrami et al.^19^ and the lithospheric thickness map of Priestley and McKenzie^5^. In Fig. 4a,b we compare the pattern of seismic anisotropy with the geodetic strain-rate fields. Figure 4c shows the pattern of azimuthal anisotropy in comparison with the plate motion vectors (blue arrows) in the ITRF2014 reference frame^20^. Figure 4d presents the correlation between the anisotropy directions and the trend of variation in the lithospheric thickness. The high degree of azimuthal correlation between the plate motion direction and azimuthal anisotropy in the Arabian Plate, eastern Turkey and the western Zagros (Fig. 4c) suggests that large-scale viscous flow in the asthenosphere is likely the dominant mechanism beneath these regions, as previously suggested^15,21–23^. On the other hand, the spatial variation in the pattern of azimuthal anisotropy across the Iranian Plateau implies a lateral change in the mantle flow and a more complex deformation history.

### Lithospheric deformation?

Anisotropy in the lithosphere can develop due to a long-term deformation history. Both pure and simple shear regimes can lead to the occurrence of anisotropy in the lithosphere^24,25^. By assuming a vertically coherent deformation, the strain-rate field estimated at the surface from GPS velocities is proposed to represent the deformation across the entire lithosphere^26^. The maximum extensional and shear strain rates are taken as proxies for the pure and simple shear deformation in the lithosphere, respectively^24^. The main assumption in this hypothesis is that the geodetic strain-rate is representative of the long-term strain field of the mantle deformation^27–30^.

Simple shear along major transcurrent faults can generate pervasive anisotropic fabric in the vicinity of the fault zone if the associated deformation affects the whole lithosphere^24,25,31^. In this case, the fast direction of azimuthal anisotropy can be oriented subparallel to the strike of fault zone as it has been observed for the main continental transform faults such as the San Andreas Fault^32,33^ and the Dead Sea Fault^34^. Since a major part of the Arabia-Eurasia convergence in Iran is accommodated by transpression-type deformation along numerous strike-slip faults^2,3^, the effect of the fault-related simple shear on the development of azimuthal anisotropy in the region needs to be taken into account. Kaviani et al.^11^ attempted to explain the pattern of seismic anisotropy across the Iranian Plateau by simple shearing related to the major strike-slip fault zones. Here, we compare the anisotropy directions with the maximum shear strain rate vectors across the study area (Fig. 4a). The two orthogonal directions of the maximum shear-strain rate represent either right- or left-lateral motions along the fault zones in each region. Despite an apparent correlation between the observed azimuthal anisotropy and the maximum shear strain rate directions in some regions, the magnitude of the shear strain rate vectors suggests insignificant shearing along the strike-slip zones. This may suggest that the strike-slip fault systems in Iran do not affect the whole lithosphere which is required for the development of pervasive anisotropy in the vicinity of the shear zones. Only in eastern Iran, we observe a more significant azimuthal correlation between the anisotropy orientations and maximum shear directions with relatively large strain rates. In this region, the fast directions of azimuthal anisotropy are mainly oriented in an N–S trend subparallel to the strike of the fault systems (Fig. 2) that accommodate the relative motion between the Lut and Helmand blocks^3,35–37^. This implies that the strike-slip fault system in eastern Iran likely acts as a major plate boundary zone capable of producing pervasive anisotropy.

Previous numerical modeling studies of viscoplastic deformation^24^ suggest that mountain-parallel LPO can develop by ductile deformation (pure shear) in the uppermost mantle induced by belt-perpendicular compressive stresses. This mechanism can explain the observations of belt-parallel seismic anisotropy in some mountain belts such as the Alps and Central Asia^28^. Overall, our observations exhibit a low azimuthal correlation between the directions of horizontal extensional strain rates (pure shear) and seismic anisotropy (Fig. 4b). In regions with an apparent correlation (such as along the narrow band in central Iran where the fast polarizations are NW–SE oriented with relatively large split time), the magnitudes of the extensional strain rate vectors are very low. In the Zagros, the horizontal extensional strain rate and azimuthal anisotropy directions are almost orthogonal. Furthermore, our examination reveals that there is no clear correlation between the lithospheric thickness and the observed splitting split times for the whole study area (Figure S2). All these lines of evidence imply that the lithospheric deformation in the form of pure shear with a horizontal maximum extensional axis has likely a negligible role in the development of azimuthal anisotropy beneath the Zagros and central Iran.

If a coherent anisotropic fabric exists beneath the Zagros, the weak correlation of the observed SWS splitting with the structural trends and horizontal strain field in the Zagros most likely suggests a dominantly vertical symmetry axis of anisotropy such that a sub-vertical traveling XKS wave is less affected. Therefore, we argue that the main part of deformation in the thick mantle lithosphere beneath the Zagros is taking place as an axial shortening with a vertical main axis of deformation. The plausible axial shortening of the lithosphere implies major coupling between the crust and the underlying mantle beneath the Zagros. In other collision zones such as Central Asia, it seems that two determining factors allow for the pure-shear deformation in the mantle^28^: (1) the relatively high gravitational potential energy (due to high elevation) that does not permit for a dominantly vertical deformation and (2) the regional tectonic setting that permits for lateral ductile flow in the uppermost mantle. In contrast, while the lower elevation of the Zagros allows for a vertical deformation (due to the axial shortening), the possible lateral variation in the lithosphere structure and the limited spatial extent of the Zagros belt does not favor a lateral mantle flow and pure-shear deformation.

On the other hand, the mountain ranges of northern Iran (Kopeh-Dagh, Alborz, and Talish) are characterized by stronger azimuthal anisotropy with trends varying along the range. The belt-parallel azimuthal anisotropy in the central Alborz and Kopeh-Dagh may suggest that the observed SWS is mainly due to an anisotropic source located in the lithosphere. This indicates a major difference with the Zagros belt where the azimuthal anisotropy directions are mainly belt-orthogonal, implying a substantial structural and dynamic difference between the southern and northern collision belts in Iran.

### Keel-induced diversion of asthenospheric flow?

Assuming that the main cause of the observed anisotropy beneath the Zagros and central Iran is mantle flow, we aim to find an explanation for its relatively complex pattern. Our observation of the circular pattern of azimuthal anisotropy (Fig. 3), though at a smaller scale, is comparable to the observations in the western United States, which is ascribed to mantle flow that is locally modified by variation in the lithospheric thickness, the presence of slab segments and a lithospheric drip^38–40^. Similar observations in other regions also suggest that the interaction between different components involved in a subduction/collision system can have significant influence on the flow field^41–44^. In a segment of the African–European continental collision zone, in the region of westernmost Mediterranean, the complex pattern of azimuthal anisotropy is attributed to a toroidal mantle flow related to the subducted slab^41,44^. These studies also propose a channelized mantle flow beneath a region with a thinned lithosphere, which explains the coherent strong azimuthal anisotropy in areas away from the region of proposed toroidal flow. Miller and Becker^43^, by geodynamic modeling of shear-wave splitting observations in the Caribbean-South American Plate subduction system, explain how the interactions between subducted slabs and cratonic keels results in a deflection of the mantle flow field^43^.

**Figure 5:**
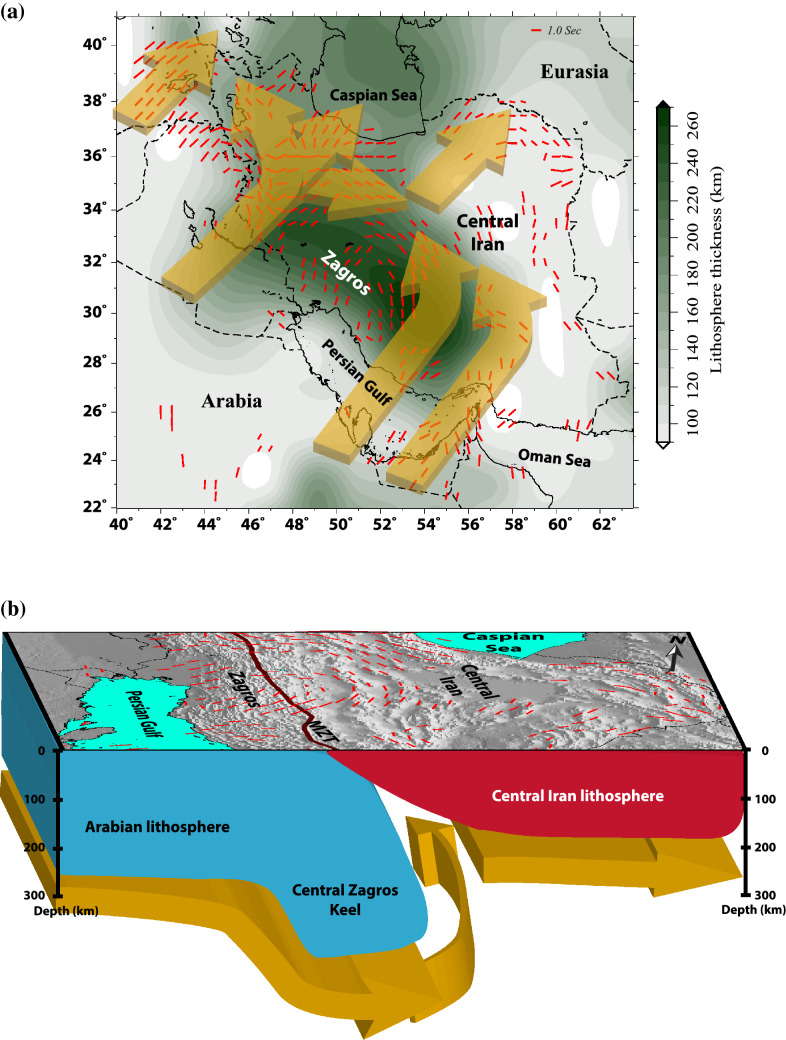
Schematic illustration of the model showing the possible circular mantle flow around the Zagros keel. (**A**) a map view and (**B**) a 3-D view of the proposed circular mantle flow. Figures are generated using the GMT^60^ and Adobe Illustrator.

The azimuthal correlation between the seismic anisotropy directions and plate-motion vectors (Fig. 4c) in eastern Turkey and western foreland of the Zagros suggests that a basal drag flow related to the plate motion is one main cause for the occurrence of anisotropic fabric in the mantle. However, the strength of the plate-driven anisotropy may be limited for the relatively slow-moving Arabian plate^45^. Faccenna et al.^46^, by numerical simulations, concluded that a large-scale mantle convection associated with the mantle upwelling underneath Afar in the south together with the Tethyan slab subduction beneath the Bitlis‐Zagros suture in the north provides the major driving forces for the kinematics of the Arabia-Anatolia-Aegean system. They suggest that the combined driving forces play the main role in the northward indenter motion of Arabia and the westward movement of Anatolia. The large-scale mantle flow, acting as a “conveyor belt”^46^, may also affect the mantle beneath the Zagros and Iranian Plateau superposing the presumably weak basal drag flow. A more recent higher resolution mantle-flow modeling by Petrunin et al.^47^ suggests that the large-scale mantle flow that emerges from the Afar upwelling has a dominant vertical component beneath the Zagros providing an additional explanation for the relatively weak observed SKS splitting time. The reduction of splitting time beneath this region can be due to a rapid shift of the regional horizontal mantle flow to a vertical flow. The proposed vertical flow can be a signature for the initiation of a lithospheric drip (due to Rayleigh–Taylor instabilities) as also proposed for some other regions such as the Great Basin in the western North America^39^. The apparent lithospheric thickening beneath the Zagros can be associated with the proposed lithospheric drip. Further assessment of this notion requires a high-resolution 3-D imaging of the Zagros lithosphere and a detailed probe of radial anisotropy, which may allow to examine the extent and strength of the proposed vertical flow. Farther east, the topography of the base of the lithosphere beneath the Zagros presents boundary conditions that cause the mantle flow to develop a complex pattern. Previous tomography studies^4,6–10^ suggest the presence of a hot (and low viscosity) mantle NE of the Zagros suture beneath the region where we observe a narrow band of the NW–SE oriented azimuthal anisotropy. The northward push of the Zagros keel^4,5^ may cause a lateral (in an NW–SE direction) flow of low viscosity material beneath this region producing a circular flow pattern (Fig. 5). The lithospheric thickness variation from the central Zagros to central Iran provides a corridor that reorganizes this lateral mantle flow. The alignment of the azimuthal anisotropy sub-parallel to the lithospheric thickness contour lines (Fig. 4d) provides additional evidence in favor of this hypothesis. An NW–SE extension in the lithosphere may also produce LPO that can be sub-parallel to the flow in the underlying asthenosphere causing higher split times. However, as mentioned above, the surface strain field suggests an insignificant lithospheric extension in this region. Further NW, at the location of the NW limit of the Zagros keel, we observe a very complex pattern of anisotropy that can be indicative of a toroidal component of flow, likely implying a sharp boundary of the Zagros keel. This area extends northward with an NNW–SSE anisotropy orientation that serves as a transition zone between the simple pattern region in eastern Turkey to the more complex patterns in the Zagros and central Iran. This transition may be associated with a major lateral discontinuity between the Zagros keel and the mantle lithosphere beneath NW Iran. The discontinuous nature of the Arabian lithosphere beneath Eurasia may have been inherited from the segmented structure of the Tethyan slab as seen by seismic images^9,10,48–50^ and the diachronous nature of the tectonic events occurred along the strike of the suture zone of the Arabia-Eurasia collision zone as suggested by geological studies^51,52^.

We summarize the idea of the proposed circular mantle flow around the Zagros keel in the interpretative model shown in Fig. 5. The circular pattern of azimuthal anisotropy at the boundary between the Zagros and central Iran suggests a circular mantle flow around the Zagros lithospheric keel. At the NW limit of the Zagros keel, a more toroidal flow pattern is likely provoked by the interaction with the NE plate-driven simple flow. This location may be related to a lateral discontinuity in the lithosphere structure of the Zagros.

## Methods

Seismic waves provide an indirect way to investigate deep lithospheric deformation and mantle flow regime via the study of seismic anisotropy. The lattice preferred orientation (LPO), as a long-term response to the cumulative strain of intrinsically anisotropic minerals (mainly olivine) is known as the main mechanism for the development of seismic anisotropy in the mantle^53–56^. In such circumstances, the fast axis of the bulk anisotropic medium is oriented sub-parallel to the maximum extension and/or flow directions in the mantle^26^. Splitting analysis of seismic shear waves is a standard approach to study seismic anisotropy in the mantle and crust. By propagating through an anisotropic medium, a shear wave splits into two components with orthogonal polarization directions and different speeds. The polarization direction (φ) of the fast component serves as a proxy for the fast symmetry axis of the anisotropic medium and thus as an indicator for the direction of maximum extension and/or mantle flow. The split time (δt) between the two components is an integral effect of the strength and extent of anisotropy along the ray path.

For each station, we jointly analyzed all available XKS waveforms, using the SplitRacer code of Reiss and Rümpker^17^. With this approach, in addition to individual splitting parameters of each XKS phase, the parameters of a one or two-layer model of anisotropy beneath the station can also be derived by joint minimization of the T-component energy^16^ of all XKS phases. A one- or two-layer model of anisotropy is accepted for each station when the total T-component energy is reduced by more than 30%. The choice of this threshold is based on the comparison between the mean values of individual measurements and parameters obtained by joint-inversion. We realized that when the energy reduction in joint-inversion is less than 30%, the one-layer parameters differ significantly from the mean value of the individual measurements, suggesting that the one-layer assumption is not valid. Since the two-layer inversion requires significant computation time, we first examined the individual splitting parameters for systematic azimuthal variation, which could be the first indicator of depth-dependent anisotropy^57,58^. Visual inspection shows that significant azimuthal variations are limited to less than 5% of stations. We also attempted to derive two-layer models at these stations in order to examine the contribution from the lithospheric and/or asthenospheric source of anisotropy. Since this two-layer (four-parameter) inversion is highly non-unique^59^, we fixed the fast direction of the lower and/or upper layer of the model (to be parallel to the plate motion and extensional strain rate directions, respectively). Our examination revealed that at these stations, a two-layer model neither leads to a better T-component energy reduction of the XKS waveform nor improves the fit of the azimuthal variation of individual splitting parameters. We conclude that in the main part of the study region, the observed azimuthal anisotropy can be attributed to a single anisotropic layer dominated by either asthenospheric flow or lithospheric deformation. However, we cannot completely rule out the cases where the two layers of anisotropy are subparallel or nearly orthogonal.

To better identify the characteristic pattern of anisotropy on the maps, we resample our observations at grid points separated by 1° in both longitude and latitude. The splitting parameters (φ and δt) at each node of the grid represent a weighted mean of all observations within the radius of the first Fresnel zone as calculated at a depth of 150 km for an SKS phase of 10 s wavelength. In this averaging scheme, the one-layer splitting parameter at each single station is linearly weighted according to its distance from the node. No value is assigned to a node if no observation occurs within the Fresnel zone.

## Supplementary Information

Supplementary Information 1.

Supplementary Information 2.

Supplementary Information 3.

Supplementary Information 4.

Supplementary Information 5.

## Data Availability

The data from the permanent stations in Iran were provided by the Iranian Seismological Center (Institute of Geophysics, University of Tehran) and International Institute of Earthquake Engineering and Seismology (IIEES). Data from the eastern Turkey was included from Kandilli Observatory Digital Broadband Seismic Network (10.7914/SN/KO, Kandilli Observatory and Earthquake Research Institute, Bosphorus Univ. (2001)). The data from temporary stations and global permanent stations and few global permanent stations was downloaded via the facilities of IRIS Data Services and specifically the IRIS Data Management Center. IRIS Data Services are funded through the Seismological Facilities for the Advancement of Geoscience and EarthScope (SAGE) Proposal of the National Science Foundation under cooperative agreement EAR1261681. This study was partially supported by the German Research Foundation (DFG) through research grants to AK and MM. The maps shown in Figs. 1, 2, 3, 4 were generated using the Generic Mapping Tools (GMT)^60^.
