# Supplementary material for: Mantle-flow diversion beneath the Iranian plateau induced by Zagros’ lithospheric keel
Source: Sci Rep. 2021 Feb 2;11:2848. doi: 10.1038/s41598-021-81541-9 (PMC7854601; doi:10.1038/s41598-021-81541-9)
Supplement: Supplementary file 1 — Supplementary Information 1. [file 41598_2021_81541_MOESM1_ESM.docx]

**Supplementary Material**

**Mantle-flow diversion beneath the Iranian plateau induced by Zagros’ lithospheric keel**

Ayoub Kaviani^1^, Meysam Mahmoodabadi^1,2^, Georg Rümpker^1^, Simone Pilia^3^, Mohammad Tatar^2^, Faramarz Nilfouroushan^4,5^, Farzam Yamini-Fard^2^, Ali Moradi^6^, and Mohammed Y. Ali^7^

^1^ Institute of Geosciences, Goethe University, Frankfurt, Germany

^2^ International Institute of Earthquake Engineering and Seismology, Tehran, Iran

^3^ Department of Earth Sciences-Bullard Labs, University of Cambridge, Cambridge, UK

^4^ Faculty of Engineering and Sustainable Development (ATM), University of Gävle, Sweden

^5^ Department of Geodetic Infrastructure, Lantmäteriet, Gävle, Sweden

^6^ Institute of Geophysics, University of Tehran, Tehran, Iran

^7^ Department of Earth Sciences, Khalifa University of Science and Technology, Abu Dhabi, UAE

The supplementary material includes two figures S1 & S2 and two tables Table 1 & Table 2.

*Figure S1: Individual SKS splitting measurements plotted at piercing point locations corresponding to depths a) 100km and b) 200km. The background shear-wave tomography maps are after Priestley and McKenzie (2013). The thick black line approximately delineates the surface trace of the boundary between the Arabian and Eurasian plates.*

*Figure S2: Split delay times vs. lithospheric thickness*

*Table 1: The list of parameters of the one-layer model as obtained by joint-inversion for each station*

*Table 2: The list of individual splitting parameters obtained by processing of each single XKS phase at each station*
