# Supplementary material for: Mantle-flow diversion beneath the Iranian plateau induced by Zagros’ lithospheric keel
Source: Sci Rep. 2021 Feb 2;11:2848. doi: 10.1038/s41598-021-81541-9 (PMC7854601; doi:10.1038/s41598-021-81541-9)
Supplement: Supplementary file 2 — Supplementary Information 2. [file 41598_2021_81541_MOESM2_ESM.pdf]

a)

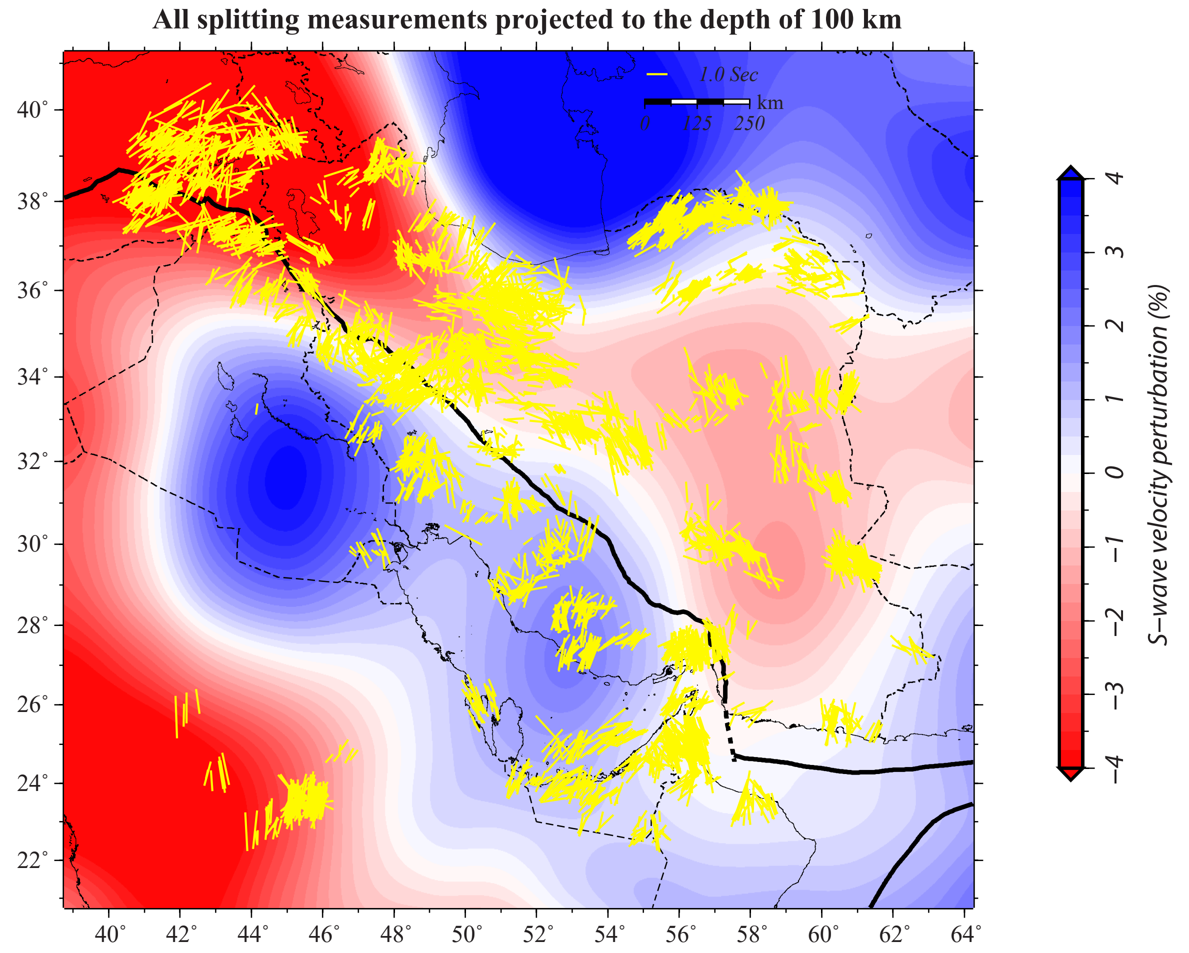
All splitting measurements projected to the depth of 100 km
1.0 Sec
km
0
125
250
40°
38°
36°
34°
32°
30°
28°
26°
24°
22°
40°
42°
44°
46°
48°
50°
52°
54°
56°
58°
60°
62°
64°
S-wave velocity perturbation (%)
4
3
2
1
0
−1
−2
−3
−4

b)

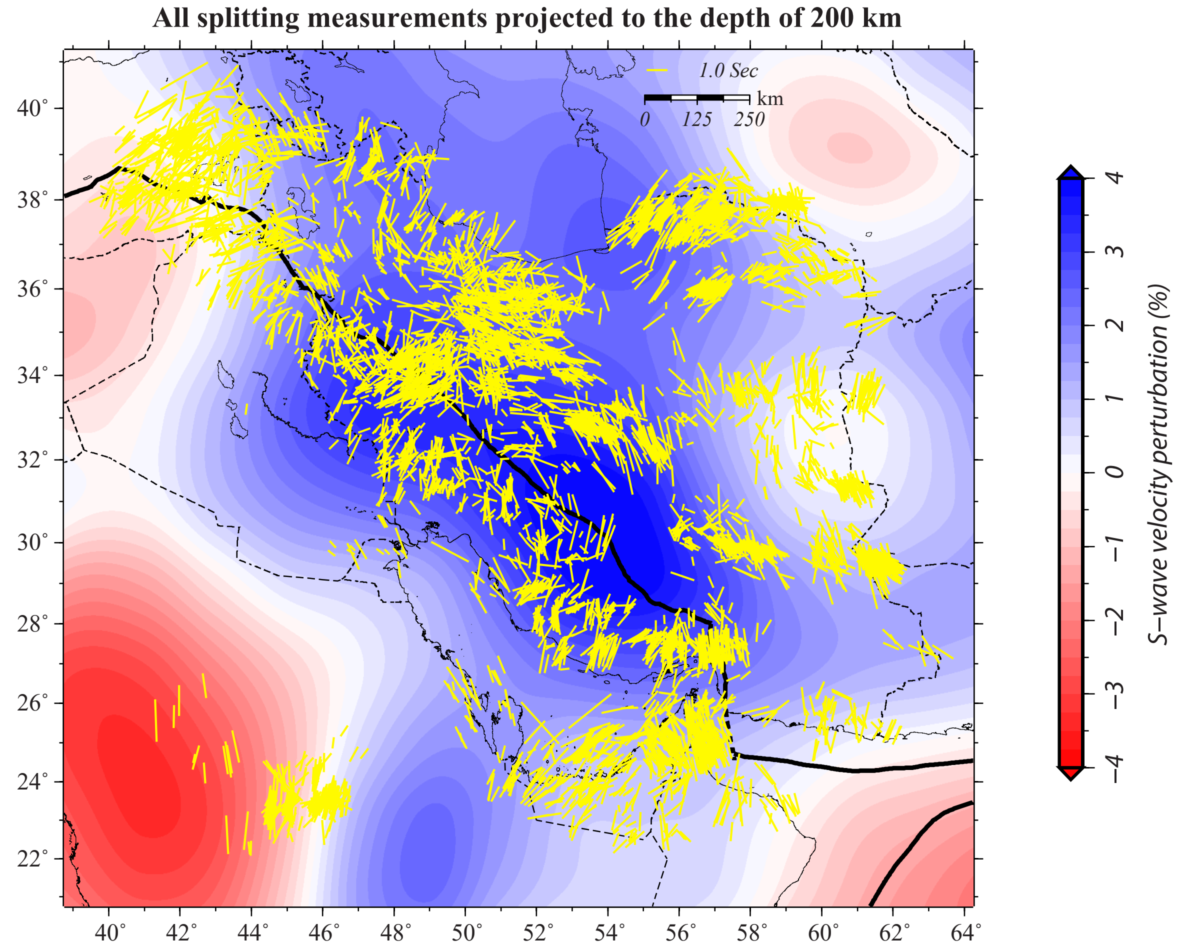
All splitting measurements projected to the depth of 200 km
1.0 Sec
km
0
125
250
40°
38°
36°
34°
32°
30°
28°
26°
24°
22°
40°
42°
44°
46°
48°
50°
52°
54°
56°
58°
60°
62°
64°
S-wave velocity perturbation (%)
4
3
2
1
0
−1
−2
−3
−4
