# Supplementary figures and images for: Mantle-flow diversion beneath the Iranian plateau induced by Zagros’ lithospheric keel
Source: Sci Rep. 2021 Feb 2;11:2848. doi: 10.1038/s41598-021-81541-9 (PMC7854601; doi:10.1038/s41598-021-81541-9)

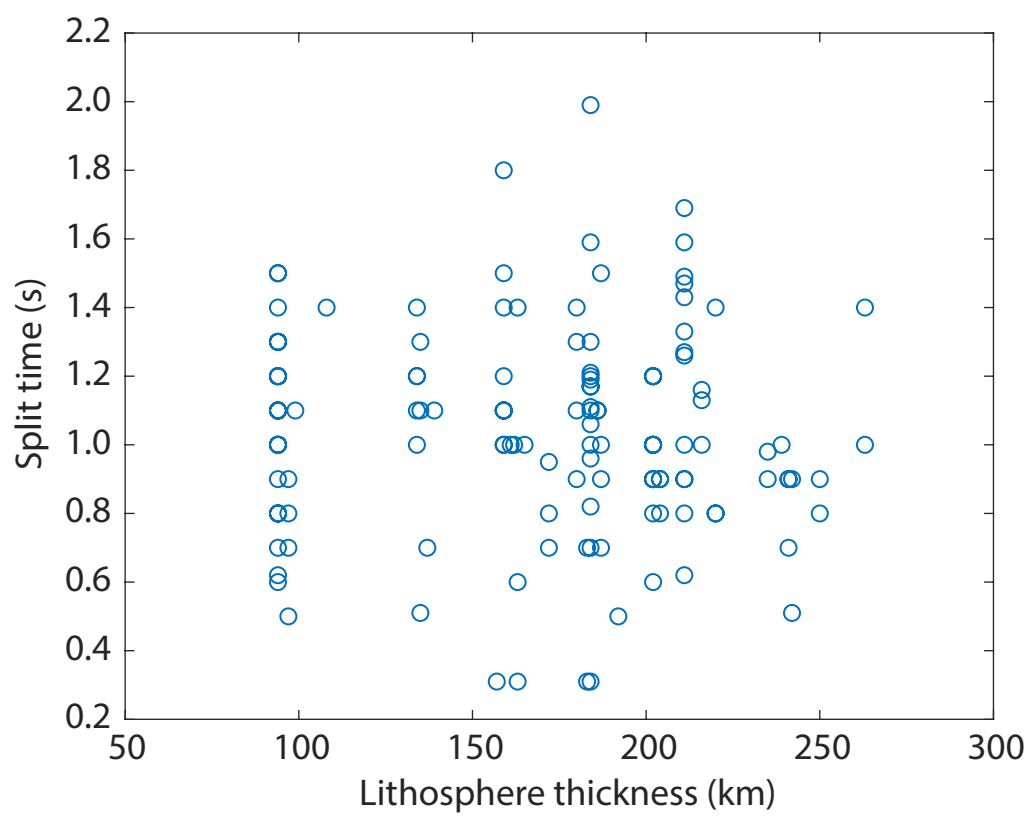

Supplement: Supplementary file 3 — Supplementary Information 3. [file 41598_2021_81541_MOESM3_ESM.pdf]
